# Household Clustering of High-Risk Contacts in Smear-Positive TB Patient Families: Evidence for Hotspot Households and Risk Stratification in Rural Eastern Cape

**DOI:** 10.3390/ijerph22121823

**Published:** 2025-12-05

**Authors:** Hloniphani Guma, Ntandazo Dlatu, Wezile Wilson Chitha, Teke Apalata, Lindiwe Modest Faye

**Affiliations:** 1 Department of Laboratory Medicine and Pathology, Faculty of Medicine and Health Sciences, Walter Sisulu University, Mthatha 5099, South Africa; 221642684@mywsu.ac.za (H.G.); tapalata@wsu.ac.za (T.A.); 2 Walter Sisulu Institute for Clinical Governance, Healthcare Administration, Department of Public Health, Faculty of Medicine and Health Sciences, Walter Sisulu University, Mthatha 5099, South Africa; ndlatu@wsu.ac.za (N.D.); wchitha@wsu.ac.za (W.W.C.)

**Keywords:** tuberculosis, household contacts, high-risk families, contact investigation, risk stratification, machine learning, clinical governance, community-engaged education

## Abstract

**Highlights:**

**Public health relevance—How does this work relate to a public health issue?**

- Household contacts of smear-positive TB patients, particularly children and individuals in crowded homes, face elevated risk of TB infection and disease progression.
- Despite WHO recommendations, implementation of contact screening and TB preventive therapy remains limited in rural high-burden settings.

**Public health significance—Why is this work of significance to public health?**

- This study identifies key demographic and household predictors such as young age and large household size linked to TB risk, enabling more effective targeting of interventions.
- The integration of machine learning models enhances risk stratification and improves efficiency in TB control efforts in resource-constrained environments.

**Public health implications—What are the key implications or messages for practitioners, policy makers and/or researchers in public health?**

- Data-driven tools can support the prioritization of high-risk households for contact tracing, TB preventive therapy, and follow-up care.
- Strengthening family-centered strategies and community-engaged education is critical to closing TB prevention gaps in underserved areas.

**Abstract:**

Background: Household contacts of smear-positive tuberculosis (TB) patients face an elevated risk of infection and disease progression, particularly young children and individuals living in overcrowded households. Despite WHO recommendations for systematic contact screening and provision of TB preventive therapy (TPT), implementation remains suboptimal in high-burden rural areas. This study aimed to develop a practical framework for identifying and prioritizing high-risk families by examining demographic predictors, household clustering, and machine learning-based risk models. Methods: A total of 437 household contacts linked to smear-positive index cases were assessed and classified as high or low risk. Statistical analyses included descriptive measures, χ^2^ tests, Z-tests for age-group differences, and multivariable logistic regression. Household-level vulnerability patterns were explored using network visualizations, clustered heatmaps, and risk-ranking charts. Three machine learning models, logistic regression, random forest, and gradient boosting, were trained using demographic and household variables with 5-fold cross-validation and an 80/20 hold-out test split. Model performance was evaluated using the AUROC, AUPRC, accuracy, F1-score, calibration curves, and decision curve analysis. Results: Of the 437 contacts, 290 (66.4%) were classified as high risk. A younger age was strongly associated with high-risk status (χ^2^ = 16.61, *p* = 0.005), with children aged 0–4 years being significantly more likely to be in a high-risk category (Z = 2.706). Gender showed no significant association (*p* = 0.523). Logistic regression identified younger age (aOR = 2.41, 95% CI: 1.48–3.94) and larger household size (aOR = 1.12 per additional member, 95% CI: 1.01–1.25) as independent predictors of the outcome. Visual analytics revealed apparent clustering of high-risk individuals within “hotspot families,” enabling prioritization through composite risk scores. Gradient boosting achieved the strongest performance (AUROC = 0.65; AUPRC = 0.76), with acceptable calibration (Brier score = 0.21) and a positive net clinical benefit in the decision curve analysis. Conclusions: TB risk is highly clustered at the household level, with large families and young children carrying disproportionate vulnerability. Combining demographic risk assessment, household-level visualization, and predictive modeling provides a practical, data-driven approach to prioritizing households during contact investigation. These findings support the WHO’s family-centered strategy and underscore the need to strengthen clinical governance and community-engaged education to optimize TB prevention in resource-limited rural settings.

## 1. Introduction

Tuberculosis (TB) is a chronic airborne infectious disease caused by *Mycobacterium tuberculosis*, a slow-growing, acid-fast bacillus within the *Mycobacterium tuberculosis* complex [1,2]. Its lipid-rich, mycolic acid-dense cell wall confers intrinsic resistance to many disinfectants and environmental stresses, enabling prolonged survival in aerosolized droplets [2]. Transmission occurs primarily through the inhalation of infectious aerosols generated by individuals with smear-positive pulmonary tuberculosis, allowing the bacilli to reach the alveoli, where they are phagocytosed by macrophages [2]. *Mycobacterium tuberculosis* can persist intracellularly and evade immune clearance, leading to latent infection and potential progression to active disease, particularly among young children and immunocompromised individuals [2,3]. These microbiological and pathogenic features underpin the intense transmission dynamics observed in high-burden settings. Tuberculosis remains a leading cause of morbidity and mortality worldwide, with an estimated 10.6 million new cases reported in 2022, of which more than a quarter occurred in Africa [1,2]. Transmission is particularly intense in resource-limited settings where overcrowding, delayed diagnosis, and inadequate preventive measures facilitate the spread of infection. Household contacts of smear-positive index cases are at exceptionally high risk, as prolonged exposure within the home significantly increases the likelihood of TB infection and disease progression [3,4,5]. Children and other vulnerable members are disproportionately affected, with the risk of developing active TB following infection estimated to be highest in the first two years of exposure [6,7].

Despite WHO recommendations that all household contacts, particularly children under five and people living with HIV, should be systematically screened and offered TB preventive therapy (TPT), implementation remains suboptimal in high-burden regions such as South Africa [8,9,10]. Barriers include limited health system capacity, underutilization of contact investigation tools, and a lack of data-driven approaches to prioritize households most in need [11,12]. Traditional screening strategies often treat households as collections of individuals, overlooking clustering of risk at the family level [13,14]. In response, innovative analytic approaches are required to identify better “hotspot families” and guide resource allocation. Household-level visualization, clustering, and predictive modeling can complement standard epidemiological methods by highlighting demographic and structural factors, such as age distribution, household size, and risk heterogeneity, that drive vulnerability [15,16]. Such approaches align with the principles of clinical governance and community-engaged TB education, offering practical strategies to strengthen preventive therapy coverage in rural, resource-constrained settings [17,18].

In this study, we focused on households of smear-positive pulmonary TB (PTB) index patients because this group represents the highest transmission risk and is therefore prioritized in South Africa’s national TB contact investigation guidelines [17]. Smear-positive individuals are the most infectious and contribute disproportionately to household and community spread, making them the most operationally appropriate group for identifying high-risk contacts in resource-limited settings. Other diagnostic approaches, such as the tuberculin skin test (TST), interferon-gamma release assays (IGRAs), or IFN-γ/IL-2 release assays, were not used because they are not routinely available at the primary healthcare level in the study district and are constrained by cost, supply interruptions, and limited laboratory capacity. In addition, this analysis focused on evaluating household-level risk patterns using routinely collected program data rather than diagnosing latent TB infection. The use of smear-positive index cases aligns with national practice and reflects the real-world context of TB control in rural South Africa. Accordingly, this study analyzed household contact data from newly diagnosed smear-positive TB patients to: (i) describe demographic and risk patterns among contacts; (ii) identify household-level clustering of vulnerability using advanced visualization techniques; and (iii) evaluate predictive models to support prioritization of preventive interventions.

## 2. Methods

### 2.1. Study Design and Population

This study employed a retrospective analysis of household contact data from newly diagnosed smear-positive pulmonary TB patients in high-burden, resource-limited rural communities of the King Sabata Dalindyebo (KSD) Municipality in the Eastern Cape Province, South Africa. The setting is characterized by overcrowded living conditions that facilitate the transmission of TB within households. Data were abstracted from routine program contact registers, anonymized, and organized by defining each household according to a single index case and all close contacts linked to that individual. A formal sample size calculation was not conducted because the study utilized the complete set of routinely collected household contact investigation records available for the defined study period. All eligible smear-positive index cases and their documented contacts were included, ensuring complete population coverage rather than a sampled dataset.

Smear microscopy, the frontline diagnostic tool at primary healthcare facilities in this district, is the primary criterion used by the South African National TB Programme to initiate household contact investigation, as smear-positive cases carry the highest transmission risk. Gold-standard diagnostics such as culture or molecular assays (e.g., Xpert MTB/RIF) were not consistently available for all index cases due to resource constraints. As the aim of this study was to examine household-level risk patterns rather than evaluate diagnostic accuracy, comparison of smear results with a gold-standard test was not undertaken.

Exclusion criteria were limited and applied to ensure data completeness and integrity. Index cases were excluded if they had no recorded household contacts, if records lacked identifiers necessary for linkage, or if contacts were missing essential variables required for analysis. Non-smear-positive TB cases were also excluded, as the study specifically focused on patients with a high transmission index, in accordance with national guidelines.

### 2.2. Variables and Outcome

The primary outcome was the TB screening classification (High risk vs. Low risk). Predictor variables included continuous and categorical age (in years), gender (male/female), and household size (number of contacts). For stratified analysis, age was categorized into three groups: child (<15 years), youth (15−24 years), and adult (≥25 years).

### 2.3. Statistical Analysis

#### 2.3.1. Descriptive and Inferential Statistics

Data were summarized using standard measures (frequencies, proportions, means, SD). Bivariate associations between the screening outcome and categorical predictors were assessed using χ^2^ or Fisher’s exact tests. Odds Ratios (OR) with 95% Confidence Intervals (CI) were calculated for categorical comparisons. Two-proportion Z-tests were used to compare age-group distributions between high-risk and low-risk household contacts, with statistical significance set at *p* < 0.05.

#### 2.3.2. Multivariable Regression

A logistic regression model was fitted to determine independent associations with the high-risk screening outcome, adjusting for age, gender, and household size. Adjusted Odds Ratios (aOR) with 95% CI were reported. Model fit was evaluated using the Hosmer–Lemeshow test and pseudo-R2 statistics.

#### 2.3.3. Machine Learning and Model Evaluation

To enhance prediction accuracy, three machine learning classifiers, logistic regression, random forest, and gradient boosting, were trained. Preprocessing included median imputation for missing age values, one-hot encoding of categorical variables, and standardization of numeric predictors. Model performance was evaluated using 5-fold stratified cross-validation and a 20% hold-out test set, with results reported as AUROC, AUPRC, accuracy, and F1-score. These findings were supported by ROC and precision–recall curves, feature-importance plots, confusion matrices, calibration curves (Brier score), and decision curve analysis.

To generate the projected reduction in high-risk contacts, this modeling framework was extended to simulate an intervention scenario incorporating strengthened clinical governance (improved screening, structured follow-up, and timely referral) and community-engaged education (enhanced TB knowledge, earlier care-seeking, and preventive practices). For each household contact, the model recalculated the probability of remaining high-risk versus shifting to a lower-risk category based on these modified behavioral and programmatic assumptions.

#### 2.3.4. Visualization and Clustering

To illustrate risk clustering and aid prioritization, several visualization techniques were applied:

- Network Diagrams: Constructed to link families and screening outcomes, using edge thickness to reflect contact counts.
- Circular Network Plots: Provided a concise overview of risk patterns across the top 20 families.
- Heatmaps with Hierarchical Clustering: Integrated household size, mean age, gender distribution, and screening outcomes to identify “hotspot families” with similar vulnerability profiles.
- Risk Ranking Bar Charts: Developed using a composite score (0–1 scale) that integrated household size, age structure, and the proportion of high-risk contacts to prioritize the most vulnerable families for intervention.

### 2.4. Ethics Approval

This analysis utilized anonymized programmatic data. Ethics clearance was secured from WSU HREC (105/2025, 9 July 2025) and EC_202507_030 (11 July 2025), adhering to all national guidelines for secondary data analysis.

## 3. Results

Among 437 household contacts of smear-positive TB patients, age distribution was significantly associated with screening outcomes (χ^2^ = 16.61, df = 5, *p* = 0.005). Younger children were disproportionately classified as high risk: 85% of those aged 0–4 years and 71% of those aged 5–14 years were identified as high risk compared to only 56% in the 25–44 age group. In contrast, older adults (≥65 years) were less frequently classified as high risk (79%). Gender was not significantly associated with screening results (χ^2^ = 0.41, df = 1, *p* = 0.523), with both males (68%) and females (65%) showing similar proportions of high-risk outcomes. Screening status also showed no significant differences (χ^2^ = 1.54, df = 1, *p* = 0.215), as nearly all contacts were screened. These findings emphasize that age is the primary demographic predictor of high-risk classification, underscoring the heightened vulnerability of children within TB-exposed households as illustrated in Table 1.

For each age group, the Z-test compares:

$$p_{1}=\frac{\mathrm{High}\;\mathrm{count}}{290},\;p_{2}=\frac{\mathrm{Low}\;\mathrm{count}}{147}$$

Using the high-risk total (*n*_1_ = 290) and low-risk total (*n*_2_ = 147), two-proportion Z-tests were performed for each age group by comparing the proportion of individuals in the high-risk group (*p*_1_ = High count/290) with the corresponding proportion in the low-risk group (*p*_2_ = Low count/147). These are presented in Table 2. 

**Figure 1:**
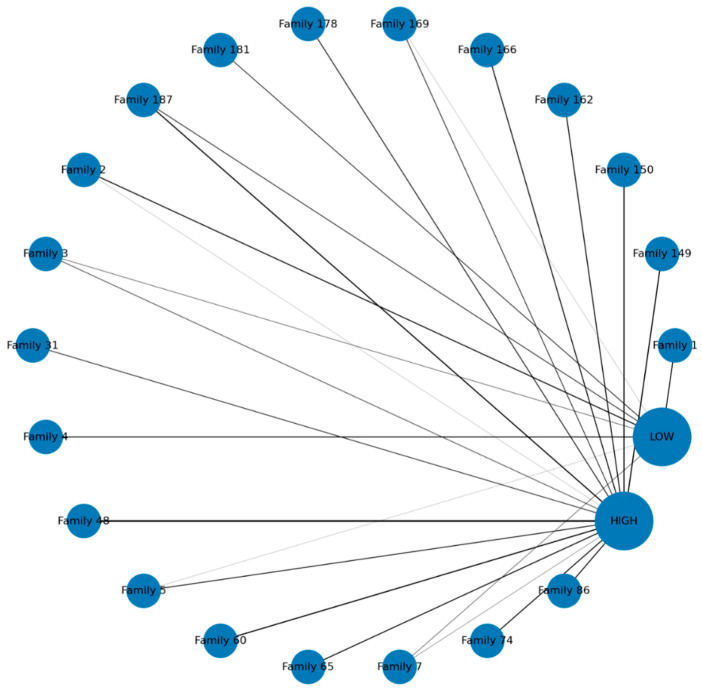
Screening results of the top 20 families.

**Figure 2:**
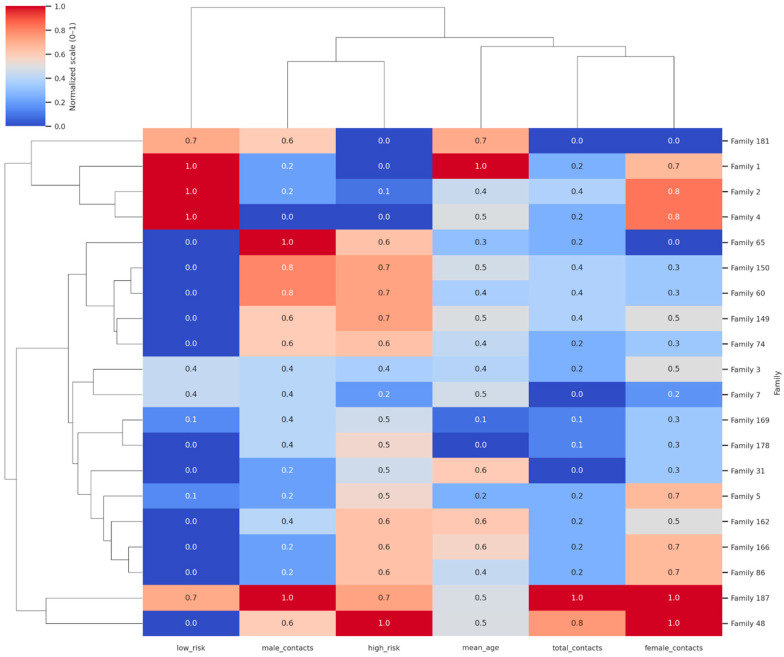
Clustered heatmap of the top 20 families of smear-positive TB index cases, grouped by demographics and screening outcomes.

**Figure 3:**
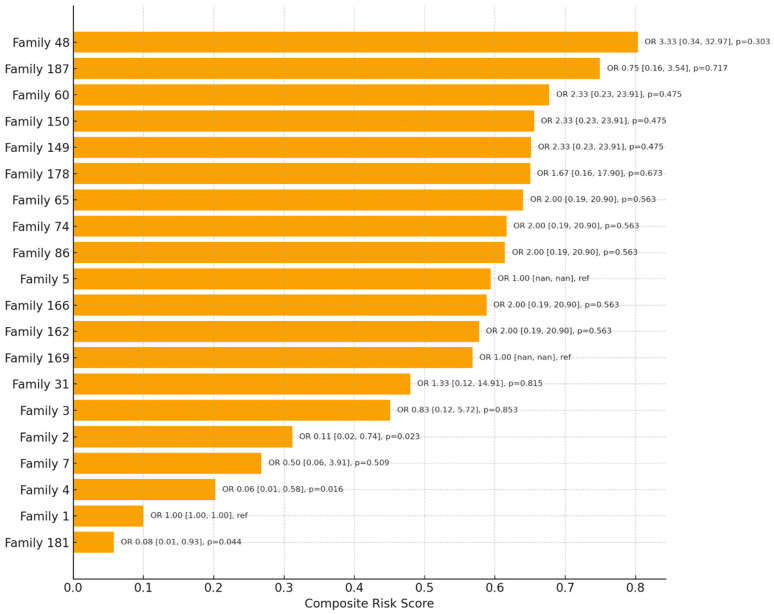
Risk ranking with family level OR.

**Figure 4:**
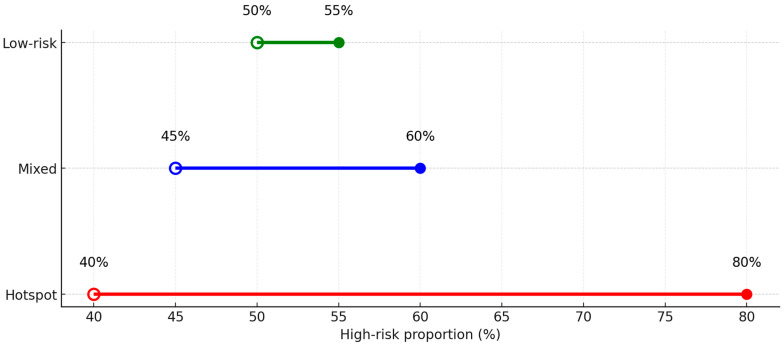
Clustered dumbbell plot showing changes in high-risk household contacts before and after governance and education interventions.

**Figure 5:**
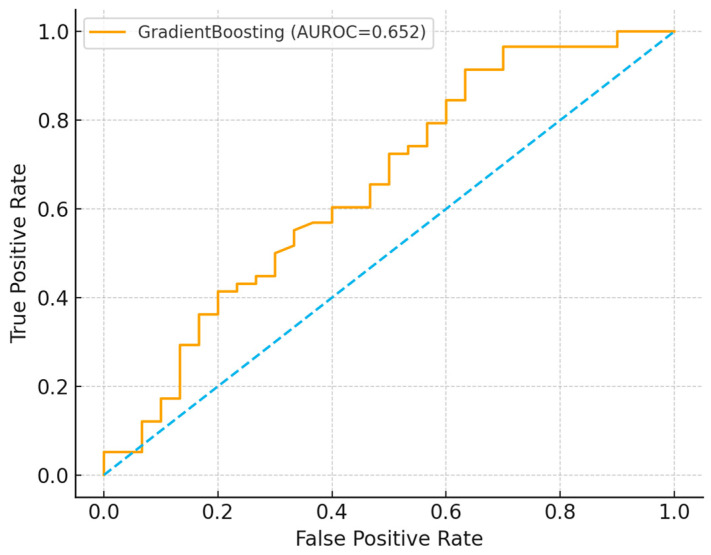
Receiver operating characteristic (ROC) curve of the best-performing gradient boosting model on the 20% hold-out test set (AUROC = 0.65), demonstrating moderate discrimination between high- and low-risk household contacts. The blue line represents the ROC curve of the model across all classification thresholds, illustrating the trade-off between sensitivity and specificity. The diagonal grey reference line (not shown) indicates no discriminatory ability (AUROC = 0.50), and the model’s curve above this line confirms performance better than chance.

**Figure 6:**
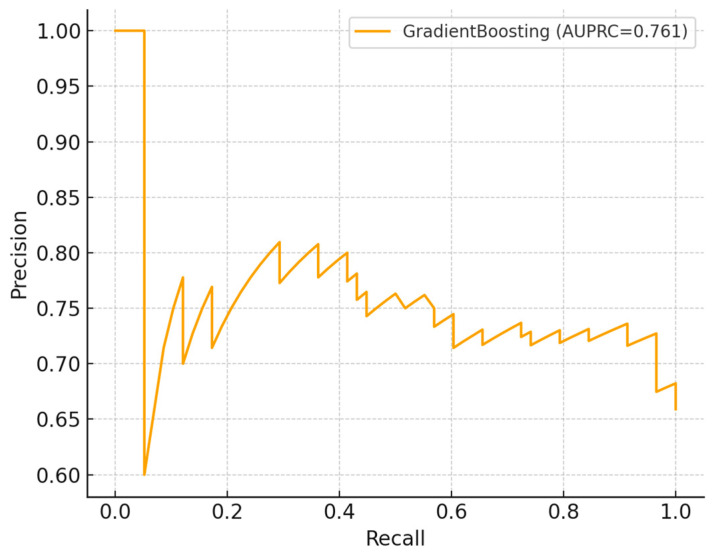
Precision–recall (PR) curve of the gradient boosting model on the hold-out set (AUPRC = 0.76), showing the ability to prioritize high-risk individuals despite class imbalance.

**Figure 7:**
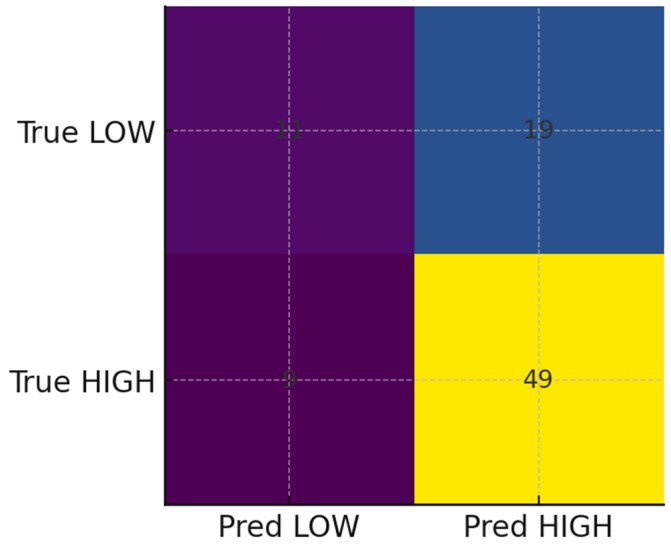
Confusion matrix at the 0.5 probability threshold for the gradient boosting model on the hold-out set, illustrating high sensitivity for high-risk contacts alongside some false positives.

**Figure 8:**
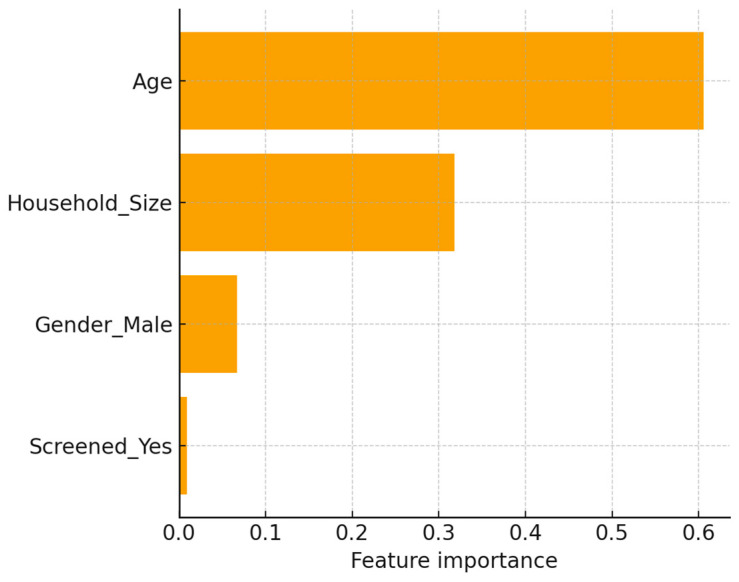
Feature importance plot from the best-performing tree-based model, highlighting age and household size as the strongest predictors of high-risk classification, with minimal contribution from gender and screening status.

**Figure 9:**
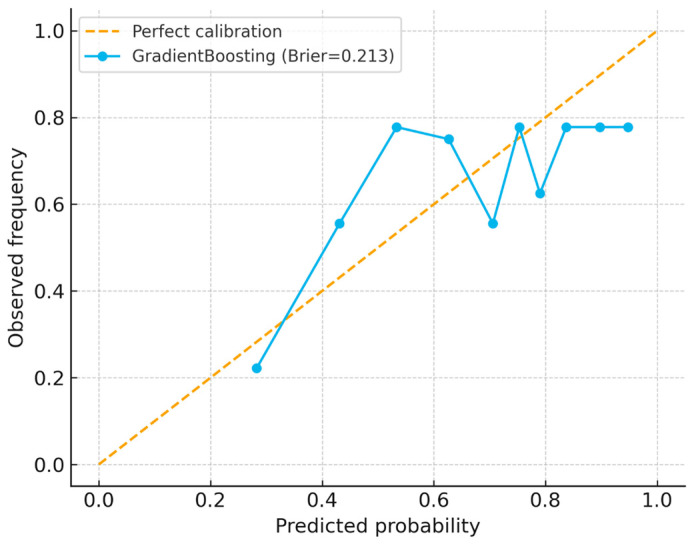
Calibration curve with Brier score (0.21), showing moderate alignment between predicted probabilities and observed outcomes, with slight underestimation at higher risk levels.

**Figure 10:**
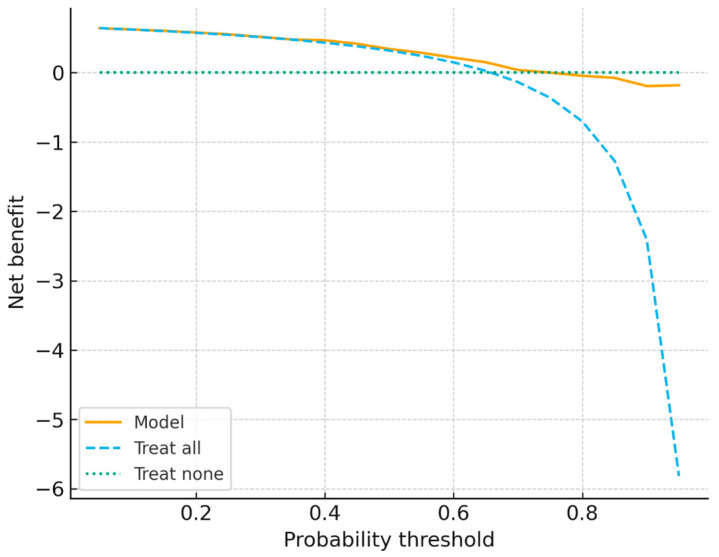
Decision curve analysis comparing the model with treat-all and treat-none strategies. The model yielded a greater net clinical benefit within probability thresholds of 0.2–0.6, supporting its utility for prioritizing preventive therapy.

The analysis showed statistically significant differences (|Z| > 1.96) in two age categories: children aged 0–4 years were significantly more represented in the high-risk group (Z = 2.706), while adults aged 25–44 years were significantly more represented in the low-risk group (Z = −2.662). No significant differences (|Z| < 1.96) were observed in the 5–14, 15–24, 45–64, or 65+ age groups, confirming that the 45–64 group displayed comparable proportions across risk classifications. These findings support the visual patterns shown in Figure 1, Figure 2, Figure 3, Figure 4, Figure 5, Figure 6, Figure 7, Figure 8, Figure 9 and Figure 10, highlighting the age-related heterogeneity in household TB risk. 

Percentages are calculated within each screening category (HIGH/LOW). χ^2^ values represent Chi-square tests of independence with corresponding degrees of freedom. A *p*-value < 0.05 was considered statistically significant. In this analysis, only the age group showed a significant association with screening outcome, indicating that younger children were more likely to be classified as high risk.

The circular network visualization in Figure 1 illustrates the distribution of TB screening outcomes across the top 20 families of smear-positive index cases. A blue node represents each family. Edges connect families to outcomes, with thickness proportional to the number of contacts in that category. The visualization demonstrates substantial clustering of high-risk contacts, as many families show thick connections to the HIGH node, indicating that vulnerability tends to occur at the household level rather than in isolated individuals. Conversely, some families are predominantly connected to the LOW node, reflecting relatively low-risk environments, while others exhibit mixed connections, suggesting heterogeneity within households. This balance of outcomes may reflect differences in household age structure, prior exposure, or the effectiveness of preventive interventions. Notably, the network highlights “hotspot families” with large numbers of high-risk contacts, providing a clear basis for prioritizing targeted interventions such as contact tracing, TB preventive therapy (TPT), and enhanced follow-up.

### Machine Learning Analysis of TB Household Contacts

We applied logistic regression, random forest, and gradient boosting models to predict high-risk TB screening outcomes among household contacts using age, gender, screening status, and household size as predictors. Data preprocessing included median imputation for missing ages, one-hot encoding for categorical variables, and standardization of numeric features. Model performance was evaluated with 5-fold stratified cross-validation (AUROC, AUPRC, Accuracy, F1) and an independent 80/20 hold-out test set for diagnostic evaluation.

In cross-validation (Table 2), gradient boosting performed best, achieving a mean AUROC of 0.65 and AUPRC of 0.76, outperforming logistic regression and random forest. On the hold-out set, the ROC curve (Figure 5) demonstrated moderate discrimination (AUROC = 0.65), while the precision–recall curve (Figure 6) indicated reasonable prioritization of high-risk individuals (AUPRC = 0.76). The confusion matrix (Figure 7) showed strong recall for high-risk contacts, balanced by a moderate number of false positives. Feature importance analysis (Figure 8) highlighted younger age and larger household size as the strongest predictors, with gender and screening status contributing minimally.

Calibration analysis (Figure 9) revealed moderate probability alignment, with a Brier score of 0.21 and slight underestimation at higher predicted risk levels. Decision curve analysis (Figure 10) revealed greater net clinical benefit of the model compared to treat-all or treat-none strategies within probability thresholds of 0.2–0.6, supporting its potential utility for prioritizing preventive therapy in high-burden households.

Overall, the models demonstrated modest discrimination but provided valuable risk stratification by reinforcing known epidemiological patterns (childhood vulnerability, household clustering) and identifying clinically relevant thresholds for programmatic decision-making, as shown in Table 3.

The clustered heatmap of the top 20 families in Figure 2 revealed clear grouping patterns based on demographic structure and screening outcomes. Columns represent total contacts, mean age, gender distribution, and high/low-risk classifications. Color intensity reflects normalized values (0–1), with clustering highlighting household “hotspot groups” characterized by large size, younger age, and high proportions of high-risk contacts. Families with large household sizes, younger mean ages, and a high proportion of high-risk contacts tend to cluster together, highlighting distinct “hotspot” groups where transmission risk is concentrated. A second cluster comprised mixed-profile households, typically of moderate size with both high- and low-risk outcomes, reflecting heterogeneity within families. A third grouping included smaller, older households with predominantly low-risk contacts, indicating lower vulnerability. The color intensity across variables highlighted these differences, with red-shaded cells corresponding to higher normalized values (e.g., more contacts or a greater high-risk burden) and blue-shaded cells indicating lower values (e.g., an older mean age or more low-risk outcomes). This clustering analysis demonstrates how family-level characteristics converge to shape TB vulnerability, offering a framework for prioritizing interventions at the household group level rather than on an individual basis.

Using penalized logistic regression with Family 1 as the reference category, several households demonstrated markedly higher odds of high-risk classification among contacts (Figure 3). Families with exclusively high-risk outcomes, such as Family 48 (11/11 high risk), Family 150 (8/8), Family 60 (8/8), and Family 149 (8/8), showed elevated odds ratios compared to the reference. However, estimates were imprecise with wide confidence intervals due to quasi-separation of outcomes (e.g., Family 48: OR = 3.33, 95% CI: 0.34–32.97, *p* = 0.303). In contrast, some families such as Family 4 (0/7 high risk) and Family 181 (0/5 high risk) had significantly lower odds compared to the reference (Family 4: OR = 0.06, 95% CI: 0.01–0.58, *p* = 0.016; Family 181: OR = 0.08, 95% CI: 0.01–0.93, *p* = 0.044). These findings highlight a clear pattern of household-level clustering of risk, where certain families disproportionately have high-risk contacts, while others exhibit relative protection. The analysis highlights the value of family-based risk stratification in prioritizing targeted interventions, such as preventive therapy and intensified follow-up.

Predictive modeling of clustered households (Figure 4) suggests that the systematic implementation of clinical governance and community-engaged education could significantly reduce the proportion of high-risk contacts. The clustered dumbbell plot summarizes the shift in high-risk burden across the three household clusters by comparing current classifications with projected outcomes after the intervention. In the Hotspot cluster (Red), the proportion of high-risk contacts decreases substantially from 80% to 40%, reflecting a strong response to targeted governance and education strategies that are likely to enhance early detection, treatment uptake, and adherence. The Mixed cluster (Blue) shows a more moderate improvement, with high-risk contacts declining from 60% to 45%. This reduction is consistent with the heterogeneous nature of these households, where the benefits of educational and governance interventions are present but less pronounced than in Hotspot families. The Low-risk cluster (Green) exhibits the least change, decreasing slightly from 55% to 50%, consistent with their baseline stability and lower initial vulnerability. In these households, the intervention yields incremental rather than transformative improvements.

Calibration was assessed using a Brier score and a calibration curve. The best model showed moderate calibration with some underestimation at higher predicted probabilities. The Brier score provides a global measure of probabilistic accuracy, with lower values indicating a better fit.

Decision curve analysis compared the net clinical benefit of using the model across probability thresholds against treat-all and treat-none strategies. The model provided a greater net benefit than treating all or none within the threshold range of 0.2–0.6, supporting its potential role in guiding the prioritization of preventive therapy.

## 4. Discussion

This study provides new evidence on the clustering of tuberculosis (TB) risk within families of smear-positive index cases in rural Eastern Cape. We observed that children and larger households were disproportionately classified as high-risk contacts, confirming the recognized vulnerability of younger populations to TB infection and progression.

### 4.1. Risk Predictors and Vulnerable Populations

Age emerged as a strong, independent predictor, with children under 15 years nearly 2.5 times more likely to be high risk compared to adults. This aligns with international findings, such as those by Afshari et al., which showed a significantly higher infection risk for children under six in large families within hyperendemic settings [19]. Furthermore, our results reinforce WHO guidance that children under five and immunocompromised individuals must be prioritized for systematic screening and preventive therapy (TPT) [20]. The finding that the youngest age groups were disproportionately high risk is particularly concerning, as South African studies show children represent the largest reservoir of preventable TB morbidity and mortality in high-burden communities [17,18,19,20].

### 4.2. Household Clustering and Prioritization

The observed clustering pattern in our network and heatmap analyses confirms that TB vulnerability is concentrated within specific “hotspot families,” rather than being randomly distributed. This echoes the feasibility and high yield of active case finding when systematic, family-unit targeting is employed in settings such as India and Ethiopia [20,21]. Our data demonstrates the added value of analytic tools, such as risk ranking and clustering visualization, in identifying families most likely to benefit from intensified follow-up. This is supported by studies, such as those by Imsanguan et al. in Thailand, which showed that practical support (e.g., transport allowances) alongside expanded investigation criteria can markedly increase case detection coverage and yield [22].

### 4.3. Improving TB Knowledge and Practices from Healthcare Workers to Communities

Improving TB knowledge, attitudes, and practices (KAP) among both healthcare workers and communities is essential for strengthening early detection, reducing transmission, and enhancing treatment outcomes. Evidence from multiple settings demonstrates that inadequate TB literacy remains a persistent challenge at various levels of the healthcare system. Among healthcare workers, studies report substantial gaps in TB and drug-resistant TB knowledge, as well as weak attitudes and suboptimal infection-control practices, despite their high occupational risk [23]. In this group, limited training, misconceptions regarding diagnostic tools, and poor understanding of treatment duration have been identified as factors contributing to inconsistent clinical practice [23]. Strengthening structured TB training for healthcare personnel is therefore critical, as improved provider knowledge directly influences patient counseling, adherence support, and case-finding efforts. At the community level, similar gaps are evident. In rural Eastern Cape, more than three-quarters of residents demonstrated low to moderate TB knowledge, driven by limited formal education, misinformation, and stigma, all of which contributed to delays in seeking testing [24]. Stigma and lack of knowledge were among the most reported barriers, reinforcing the need for culturally grounded, community-led education strategies that leverage peer educators, TB survivors, and community health workers [24]. Similarly, poor knowledge and inconsistent TB-prevention practices among household contacts have been documented in China, where inadequate understanding of transmission, treatment, and preventive behaviors contributed to risky household practices such as continued close contact before treatment initiation [25]. These findings further underscore the crucial role of accessible, tailored TB education, particularly in suburban and rural settings where information barriers are most prevalent. Collectively, these studies underscore that improving TB KAP must begin with healthcare workers, who serve as the primary source of information and model of best practice for patients, and extend outward to households and communities. Strengthened governance, consistent training, and community-engaged education initiatives are therefore essential components of a comprehensive TB-control strategy.

### 4.4. Clinical and Governance Implications

A family-centered approach has significant behavioral and governance implications. Research by Khamai et al. showed that preventive behaviors among TB contacts are shaped by perceived susceptibility, self-efficacy, and health education [26]. This may partially explain why some families in our study displayed mixed high- and low-risk outcomes. This suggests that community-engaged education and even partial TPT uptake may buffer against uniform vulnerability. Strengthening clinical governance to embed household risk profiles into district-level TB programs is critical, as this would enhance accountability, optimize scarce resources, and address the structural barriers noted by Otero et al. in Peru, where gaps in TPT initiation and completion exist despite high eligibility [27,28].

### 4.5. Alignment with Global Challenges

Our findings align with international evidence linking household clustering to broader social determinants. For instance, Li et al. reported that substantial TB transmission in rural China occurred among family and social contacts, advocating for interventions that extend beyond the index household [27]. These challenges, including suboptimal TPT coverage despite a high burden, resonate deeply within South Africa. Implementing child-focused preventive strategies within robust clinical governance frameworks and community-based programs is therefore crucial to reducing transmission, closing gaps in household care, and advancing progress toward End TB targets [10].

### 4.6. Visualization and the “Hotspot Family” Phenomenon

The circular network and clustered heatmap visualizations powerfully underscore the phenomenon of TB risk clustering at the household level, consistent with evidence that transmission is concentrated within families rather than evenly distributed across communities [6]. The visualizations clearly identified “hotspot families,” forming distinct clusters characterized by larger contacts, younger mean ages, and consistently high proportions of high-risk outcomes [29]. This high-vulnerability profile is likely driven by shared intense exposure to the index case and increased crowding, which sustains higher transmission potential. Conversely, smaller households with older contacts formed a separate, predominantly low-risk cluster. The mixed-profile households observed in both visualizations highlight intrafamilial heterogeneity, influenced by factors like prior exposure, differential immune responses, or partial uptake of preventive therapy. From a programmatic perspective, the visual identification of household “hotspots” provides a clear, practical means of prioritizing limited resources for contact tracing, TPT provision, and ongoing surveillance, reinforcing the WHO′s call for family-centered contact management as a cornerstone of the End TB Strategy [30].

### 4.7. Predictive Analysis and Model Utility

The study demonstrates the potential of machine learning (ML) approaches to augment TB household contact risk stratification. The Gradient Boosting model achieved modest discrimination (AUROC and AUPRC values consistent with other studies reliant on limited demographic data) [31,32,33]. The model identified younger age and larger household size as the most prominent predictors, reinforcing well-established epidemiological patterns of elevated vulnerability in children and crowded environments [34,35,36]. Crucially, calibration and decision curve analyses indicated a net clinical benefit within a 0.2–0.6 threshold probability range, despite moderate predictive accuracy. This suggests that the models can provide actionable programmatic insights that complement existing WHO recommendations for TPT prioritization. The analysis highlights the promise of combining ML with clinical governance and community engagement to optimize the allocation of preventive interventions in resource-constrained settings.

### 4.8. Integrated Strategy for TB Control

By combining statistical prediction with visual clustering, this integrated approach highlights the transformative potential of embedding clinical governance and community-engaged education into household TB control. The objective is to shift families out of high-risk clusters, directly addressing the concentrated transmission observed in rural communities where poverty and delayed care perpetuate vulnerability. Strengthened clinical governance, achieved through systematic contact tracing and routine TPT provision, ensures high-burden households are consistently reached. Concurrently, community education builds trust, reduces stigma, and empowers early symptom recognition and treatment adherence [27]. These synergistic improvements align with the WHO End TB Strategy by prioritizing family-centered preventive therapy and supporting national goals to reduce the incidence and mortality of TB. The model illustrates how local governance and community participation can collaborate to reduce household disparities, thereby accelerating progress toward global TB elimination targets [30,31,32,33,34,35,36].

### 4.9. Recommendations

To strengthen TB control in high-burden settings, program managers should adopt a risk-stratified, family-centered approach by prioritizing “hotspot families.”

### 4.10. Prioritized Resource Allocation

- Focus on Hotspot Families: Prioritize households identified through composite risk scoring and clustering visualization (e.g., heatmaps and risk rankings) as having large size, younger age structures, and a high proportion of high-risk contacts.
- Intensified Interventions: For these highest-risk families, allocate resources for intensive contact tracing, provision of TB Preventive Therapy (TPT), and sustained follow-up. This aligns with the WHO family-centered care approach, specifically ensuring TPT for children under five and other vulnerable members.
- Stratified Monitoring: Lower-risk households, characterized by smaller size and older age structures, should receive lighter, routine monitoring, thus maximizing the efficiency of limited resources.
- Integration into Governance and Practice.
- Data-Driven Governance: Integrate household risk profiles (using tools like risk rankings and heatmaps) into district-level clinical governance systems. This improves decision-making, enhances accountability, and optimizes the efficiency of TB prevention programs.
- Actionable Framework: The study’s framework, which combines traditional statistical analysis, visualization, and predictive modeling, offers a practical guide for implementing this risk-based strategy.

## 5. Conclusions

This study confirms the significant household-level clustering of TB risk among close contacts of smear-positive index cases. Descriptive and multivariable analyses, supported by machine learning, consistently identified younger age and larger household size as the strongest independent predictors of high-risk classification.

Network visualizations, heatmaps, and risk ranking tools further validated the existence of “hotspot families” where vulnerability is concentrated, underscoring the necessity of prioritizing interventions at the family rather than the individual level. The finding that even modestly performing predictive models can provide actionable programmatic value through decision curve analysis justifies their integration into resource allocation strategies, particularly for child-dominated, high-burden households. These results strongly support the WHO′s family-centered approach to contact management and TPT. By emphasizing the integration of clinical governance and community-engaged education, this work provides an actionable, risk-based framework to strengthen prevention, improve uptake, and accelerate progress toward TB control in resource-limited rural settings.

## Figures and Tables

**Table 1:** Demographic and clinical characteristics of household contacts (N = 437).

Variable	Screening Result: HIGH	Screening Result: LOW	Total	χ^2^ (df)	*p*-Value
Age group				16.61 (5)	0.005
0–4 years	35 (12%)	6 (4%)	41 (9%)		
5–14 years	70 (24%)	29 (20%)	99 (23%)		
15–24 years	45 (16%)	30 (20%)	75 (17%)		
25–44 years	66 (23%)	51 (35%)	117 (27%)		
45–64 years	46 (16%)	24 (16%)	70 (16%)		
65+ years	27 (9%)	8 (5%)	35 (8%)		
Gender				0.41 (1)	0.523
Female	145 (50%)	79 (54%)	224 (51%)		
Male	145 (50%)	68 (46%)	213 (49%)		
Screened status				1.54 (1)	0.215
Yes	290 (100%)	145 (99%)	435 (100%)		
No	0 (0%)	2 (1%)	2 (0%)		

**Table 2:** Two-proportion Z-Test results for age-group differences between high-risk and low-risk household contacts.

Age Group	High Proportion (p1)	Low Proportion (p2)	Z-Value
0–4 years	0.1207	0.0408	2.706
5–14 years	0.2414	0.1973	1.041
15–24 years	0.1552	0.2041	−1.281
25–44 years	0.2276	0.3469	−2.662
45–64 years	0.1586	0.1633	−0.125
65+ years	0.0931	0.0544	1.408

**Table 3:** Cross-validated performance (5-fold).

Model	AUROC (Mean (SD))	AUPRC (Mean (SD))	Accuracy (Mean (SD))	F1 (Mean (SD))
Logistic Regression	0.572 (0.034)	0.716 (0.040)	0.666 (0.007)	0.799 (0.004)
Random Forest	0.632 (0.031)	0.753 (0.018)	0.634 (0.027)	0.732 (0.029)
Gradient Boosting	0.657 (0.052)	0.785 (0.042)	0.675 (0.026)	0.776 (0.024)

## Data Availability

Data are available from the corresponding author upon request.

## References

[1] Li X., Li Y., Guo L., Chen Y., Wang G., Zhang H. (2025). Tuberculosis incidence, deaths and disability-adjusted life years in children and adolescence, 1990–2021: Results from the Global Burden of Disease Study 2021. PLoS ONE.

[2] World Health Organization (2023). Global Tuberculosis Report 2023. https://www.who.int/publications/i/item/9789240083851.

[3] Velen K., Nhung N.V., Anh N.T., Cuong P.D., Hoa N.B., Cuong N.K., Dung N.H., Sy D.N., Britton W.J., Marks G.B. (2021). Risk factors for tuberculosis (TB) among Household Contacts of patients with smear-positive TB in 8 provinces of Vietnam: A nested case-control study. Clin. Infect. Dis..

[4] Krishnamoorthy Y., Ezhumalai K., Murali S., Rajaa S., Jose M., Sathishkumar A., Soundappan G., Horsburgh C., Hochberg N., Johnson W.E. (2021). Prevalence and risk factors associated with latent tuberculosis infection among household contacts of smear-positive pulmonary tuberculosis patients in South India. Trop. Med. Int. Health.

[5] Fox G.J., Johnston J.C., Nguyen T.A., Majumdar S.S., Denholm J.T., Asldurf H., Nguyen C.B., Marks G.B., Velen K. (2021). Active case-finding in contacts of people with TB. Int. J. Tuberc. Lung Dis..

[6] Martinez L., Cords O., Horsburgh C.R., Andrews J.R., Acuna-Villaorduna C., Ahuja S.D., Altet N., Augusto O., Baliashvili D., Basu S. (2020). The risk of tuberculosis in children after close exposure: A systematic review and individual-participant meta-analysis. Lancet.

[7] Laycock K.M., Enane L.A., Steenhoff A.P. (2021). Tuberculosis in adolescents and young adults: Emerging data on TB transmission and prevention among vulnerable young people. Trop. Med. Infect. Dis..

[8] Macharia E. (2021). Uptake of Tuberculosis Preventive Therapy Among Eligible Children Under Five Years in Mombasa County. Ph.D. Thesis.

[9] Chandra D.K., Moll A.P., Altice F.L., Didomizio E., Andrews L., Shenoi S.V. (2022). Structural barriers to implementing recommended tuberculosis preventive treatment in primary care clinics in rural South Africa. Glob. Public Health.

[10] World Health Organization (2020). WHO Consolidated Guidelines on Tuberculosis. Module 1: Prevention—Tuberculosis Preventive Treatment. https://apps.who.int/iris/handle/10665/331170.

[11] Aklie E.N. (2024). The Ghanaian War Against Malaria: A Geospatial Approach to Malaria and Healthcare Access in Ghana. Master’s Thesis.

[12] Nhleko P.N. (2021). Mobile Health Technology to Improve Tuberculosis Contact Tracing in Sub-Saharan Africa: A Systematic Review, 2010 to 2021. Master’s Thesis.

[13] Kerkhoff A.D., West N.S., Castro M.d.M., Branigan D., Christopher D.J., Denkinger C.M., Nhung N.V., Theron G., Worodria W., Yu C. (2023). Placing the values and preferences of people most affected by TB at the center of screening and testing: An approach for reaching the unreached. BMC Glob. Public Health.

[14] Calderwood C.J., Timire C., Mavodza C., Kavenga F., Ngwenya M., Madziva K., Fielding K., Dixon J., Ferrand R.A., Kranzer K. (2024). Beyond tuberculosis: A person-centred and rights-based approach to screening for household contacts. Lancet Glob. Health.

[15] Hamada Y. (2025). Research to Understand Multimorbidity in Households Affected by Tuberculosis. Ph.D. Thesis.

[16] Montgomery R.M. (2025). The Multifactorial Determinants of Tuberculosis Mortality: A Global Comprehensive Epidemiological Analysis and Framework for Disease Elimination. https://www.preprints.org/manuscript/202504.1304.

[17] National Department of Health, South Africa (2023). South African Tuberculosis Control Programme: Practical Guidelines.

[18] Kilale A.M., Makasi C., Majaha M., Manga C.D., Haule S., Hilary P., Kimbute O., Kitua S., Jani B., Range N. (2022). Implementing tuberculosis patient cost surveys in resource-constrained settings: Lessons from Tanzania. BMC Public Health.

[19] Afshari M., Dehmardeh A., Hoseini A., Moosazadeh M. (2023). Tuberculosis infection among children under six in contact with smear-positive cases: A study in a hyper-endemic area of Iran. J. Clin. Tuberc. Other Mycobact. Dis..

[20] Jember T., Hailu G., Wassie G.T. (2023). Assessment of family tuberculosis contact screening practice and its associated factors among pulmonary tuberculosis positive patients in South Wollo zone, Amhara region, Ethiopia. Int. J. Public Health.

[21] Chawla S., Gupta V., Gour N., Grover K., Goel P.K., Kaushal P., Singh N., Ranjan R. (2020). Active case finding of tuberculosis among household contacts of newly diagnosed tuberculosis patients: A community-based study from southern Haryana. J. Fam. Med. Prim. Care.

[22] Imsanguan W., Chiyasirinroje B., Nedsuwan S., Yanai H., Tokunaga K., Palittapongarnpim P., Murray M., Mahasirimongkol S. (2020). Contact tracing for tuberculosis, Thailand. Bull. World Health Organ..

[23] Abbas R., Salami A., Ghssein G. (2025). Knowledge, attitude, and practices of healthcare workers towards tuberculosis, multidrug-resistant tuberculosis, and extensively drug-resistant tuberculosis. Acta Microbiol. Hell..

[24] Dlatu N., Tsuro U., Faye L.M., Hosu M.C., Sineke N., Apalata P. (2024). Towards Community-Driven Tuberculosis Education: Findings from a Knowledge and Engagement Pilot Survey in the Rural Community of Eastern Cape, South Africa. Front. Public Health.

[25] Zhang Y., Wu J., Hui X., Zhang P., Xue F. (2024). Knowledge, attitude, and practice toward tuberculosis prevention and management among household contacts in Suzhou Hospital, Jiangsu province, China. Front. Public Health.

[26] Khamai N., Seangpraw K., Ong-Artborirak P. (2024). Using the Health Belief Model to Predict Tuberculosis Preventive Behaviors Among Tuberculosis Patients’ Household Contacts During the COVID-19 Pandemic in the Border Areas of Northern Thailand. J. Prev. Med. Public Health.

[27] Li M., Guo M., Peng Y., Jiang Q., Xia L., Zhong S., Qiu Y., Su X., Zhang S., Yang C. (2022). High proportion of tuberculosis transmission among social contacts in rural China: A 12-year prospective population-based genomic epidemiological study. Emerg. Microbes Infect..

[28] Otero L., Battaglioli T., Ríos J., De la Torre Z., Trocones N., Ordonezm C., Seas C., Van der Stuyft P. (2020). Contact evaluation and isoniazid preventive therapy among close and household contacts of tuberculosis patients in Lima, Peru: An analysis of routine data. Trop. Med. Int. Health.

[29] Coleman M., Martinez L., Theron G., Wood R., Marais B. (2022). Mycobacterium tuberculosis transmission in high-incidence settings—New paradigms and insights. Pathogens.

[30] World Health Organization (2022). Implementing the End TB Strategy: The Essentials, 2022 Update. https://www.who.int/publications/i/item/9789240065093.

[31] Boothe D.B. (2022). Tuberculosis Elimination in Arkansas: Modeling Incidence and Evaluation of Screening Strategy. Ph.D. Thesis.

[32] Rae J.D., Landier J., Simpson J.A., Proux S., Devine A., Maude R.J., Thu A.M., Wiladphaingern J., Kajeechiwa L., Thwin M.M. (2021). Longitudinal trends in malaria testing rates in the face of elimination in eastern Myanmar: A 7-year observational study. BMC Public Health.

[33] Ntshiqa T., Nagudi J., Hamada Y., Copas A., Stender S., Sabi I., Ntinginya E.N., Lalashowi J., Matete M., Ntshamane K. (2025). Risk Factors Associated With Tuberculosis Infection Among Household Contacts of Patients With Microbiologically Confirmed Pulmonary Tuberculosis in 3 High Tuberculosis Burden Countries. J. Infect. Dis..

[34] van Staden Q. (2022). Access to Tuberculosis Testing Among Adolescents Living with Human Immunodeficiency Virus in the Eastern Cape, South Africa: Social factors and Theoretical Considerations. Master’s Thesis.

[35] Konkor I. (2023). Understanding the Connections Between Neighborhood Environments and Ghana’s Burden of Infectious and Non-Communicable Diseases. Ph.D. Thesis.

[36] Kim S., Wu X., Hughes M.D., Upton C., Narunsky K., Mendoza-Ticona A., Khajenoori S., Gonzales P., Badal-Faesen S., Shenje J. (2022). High Prevalence of Tuberculosis Infection and Disease in Child Household Contacts of Adults With Rifampin-resistant Tuberculosis. Pediatr. Infect. Dis. J..

